# Functional Evolution of a Multigene Family: Orthologous and Paralogous Pheromone Receptor Genes in the Turnip Moth, *Agrotis segetum*

**DOI:** 10.1371/journal.pone.0077345

**Published:** 2013-10-10

**Authors:** Dan-Dan Zhang, Christer Löfstedt

**Affiliations:** Department of Biology, Lund University, Lund, Sweden; CNRS, France

## Abstract

Lepidopteran pheromone receptors (PRs), for which orthologies are evident among closely related species, provide an intriguing example of gene family evolution in terms of how new functions may arise. However, only a limited number of PRs have been functionally characterized so far and thus evolutionary scenarios suffer from elements of speculation. In this study we investigated the turnip moth *Agrotis segetum*, in which female moths produce a mixture of chemically related pheromone components that elicit specific responses from receptor cells on male antennae. We cloned nine *A. segetum* PR genes and the *Orco* gene by degenerate primer based RT-PCR. The nine PR genes, named as *AsegOR1* and *AsegOR3-10*, fall into four distinct orthologous clusters of known lepidopteran PRs, of which one contains six paralogues. The paralogues are under relaxed selective pressure, contrasting with the purifying selection on other clusters. We identified the receptors AsegOR9, AsegOR4 and AsegOR5, specific for the respective homologous pheromone components (*Z*)-5-decenyl, (*Z*)-7-dodecenyl and (*Z*)-9-tetradecenyl acetates, by two-electrode voltage clamp recording from *Xenopus laevis* oocytes co-expressing *Orco* and each PR candidate. These receptors occur in three different orthologous clusters. We also found that the six paralogues with high sequence similarity vary dramatically in ligand selectivity and sensitivity. Different from AsegOR9, AsegOR6 showed a relatively large response to the behavioural antagonist (*Z*)-5-decenol, and a small response to (*Z*)-5-decenyl acetate. AsegOR1 was broadly tuned, but most responsive to (*Z*)-5-decenyl acetate, (*Z*)-7-dodecenyl acetate and the behavioural antagonist (*Z*)-8-dodecenyl acetate. AsegOR8 and AsegOR7, which differ from AsegOR6 and AsegOR1 by 7 and 10 aa respectively, showed much lower sensitivities. AsegOR10 showed only small responses to all the tested compounds. These results suggest that new receptors arise through gene duplication, and relaxed evolutionary constraints or positive selection among paralogues allow functional divergence to occur in spite of purifying selection being the norm.

## Introduction

Sex pheromone communication in moths, which involves the production of specific sex pheromones in females and the corresponding selective detection and response by pheromone receptors (PRs) in conspecific male antennae, has long been the intriguing subject for evolutionary studies. Coordinated changes of female pheromone signal and specific male response are necessary during the evolution of the system. For pheromone biosynthesis, previous studies have shown that the pheromone production can evolve both by gene duplication followed by structural mutations in the gene coding region [1-4] and by differential regulation in the expression of a standing set of genes [5,6]. It remains a conundrum how the corresponding variation in male detection and response originates and evolves. A few recent studies suggest that gene duplication and changes in coding sequences [7,8] rather than regulation of gene expression underlie the variation in PRs and thus may enable a change in the species-specific male response [9,10]. However, as a limited number of insect PR genes have been deorphaned, the functional evolution of the receptors remains poorly understood and little is known about how sequence changes are associated with changes in ligand specificity or sensitivity.

The turnip moth, *Agrotis segetum* (Noctuidae) has evolved a complex sex pheromone communication system, which provides an excellent model to elucidate ligand-receptor relationships. *A. segetum* makes use of a sex pheromone consisting of four structurally similar sex pheromone components (*Z*)-5-decenyl, (*Z*)-5-dodecenyl, (*Z*)-7-dodecenyl and (*Z*)-9-tetradecenyl acetates (*Z*5-10:OAc, *Z*5-12:OAc, *Z*7-12:OAc, and *Z*9-14:OAc), that are specifically perceived by distinct types of sensilla trichodea in males. A detailed single-sensillum recording study demonstrated that for *Z*5-10:OAc-type sensilla (Type-1 sensilla) there are at least three subtypes, each possessing a different tuning profile to other components [11-13]. The projection of olfactory receptor neurons (ORNs) to different regions of the macroglomerular complex is also quite specific [14] but potential differences in the projection of ORN subtypes were not investigated. The alcohol, (*Z*)-5-decenol (*Z*5-10:OH), may be found in female pheromone gland extracts but acts as a behavioural antagonist. Another compound, (*Z*)-8-dodecenyl acetate (*Z*8-12:OAc) also acts as a behavioural antagonist according to wind tunnel and field experiments, but is not produced in the sex pheromone gland of females [15].

In the present study we cloned and functionally characterized *Orco* and nine PR genes from *A. segetum*, which grouped in four different orthologous clusters of known lepidopetran PRs. In addition to the identification of specific receptors for three of the pheromone components, occuring in three different clusters, we also found that the six paralogues within one expansion differ dramatically in ligand selectivity and sensitivity. Based on these findings and analysis of selection operating on the different genes, we propose a possible scenario for the functional evolution of the pheromone receptor multigene family in moths.

## Results

### Nine candidate pheromone receptor genes and *Orco* gene cloned from *A. segetum*

Based on the amino acid sequence alignment of the PRs that had been functionally identified prior to this study, several pairs of degenerate primers were designed to perform RT- PCR (Table S1) for *A. segetum*. This led to the amplification of many cDNA fragments, which were classified into nine groups according to their sequence identities. Nine pairs of primers were then designed for 5’ and 3’-RACE PCR on the basis of the sequences in each group (Table S1). By assembling sequences of cDNA fragments, with corresponding 5’ and 3’ cDNA ends, we obtained nine cDNAs putatively encoding PRs, which were later confirmed by amplification and sequencing of full length ORF cDNAs. According to the conventions of OR nomenclature and their order of discovery, we named these PR genes *AsegOR1* and *AsegOR3*-*10* (Genbank accession numbers KC526965- KC526973). ‘*AsegOR2*’ was not used to avoid confusion with *Orco*, which has been named *OR2* in other moth species. *AsegOR1* and *AsegOR3*-*10* encode proteins ranging from 424-443 amino acids in length. Six of the candidate PRs, i.e., AsegOR1 and AsegOR6-10, share high levels of amino acid sequence identity with each other (> 70 %), whereas the remaining PRs share relatively low sequence identity (~ 35-55 %) (Table 1).

**Table 1:** Percent identities of amino acid residues among the nine PR genes.

	**AsegOR1**	**AsegOR3**	**AsegOR4**	**AsegOR5**	**AsegOR6**	**AsegOR7**	**AsegOR8**	**AsegOR9**	**AsegOR10**
**AsegOR1 (432 aa)**	-	46.2%	38.0%	52.8%	**82.9%**	***97.7%***	**83.1%**	**74.2%**	***90.7%***
**AsegOR3 (435 aa)**		-	38.2%	43.2%	43.2%	46.7%	43.2%	45.2%	47.1%
**AsegOR4 (424 aa)**			-	36.1%	39.8%	39.0%	39.8%	39.0%	39.9%
**AsegOR5 (443 aa)**				-	52.4%	52.4%	52.1%	50.3%	54.0%
**AsegOR6 (430 aa)**					-	**83.1%**	***98.4%***	**71.0%**	**83.6%**
**AsegOR7 (432 aa)**						-	**83.3%**	**74.4%**	***91.4%***
**AsegOR8 (430 aa)**							-	**70.3%**	**83.8%**
**AsegOR9 (433 aa)**								-	**73.3%**
**AsegOR10 (432 aa)**									-

The percent identities of amino acid sequences were analysed by Vector NTI Advance 10 software. Sequence identities above 70% are indicated by bold, and those above 90% are indicated by bold and italic.

In addition, we cloned the *Orco* gene from *A. segetum*, which encodes a predicted protein of 473 amino acids. According to the unified nomenclature system for the insect olfactory co-receptor [16], we named this gene as *Aseg\Orco* (Genbank accession number KC526964). It shares 63.5 % amino acid identity with *Drosophila melanogaster* OR83b, and 85.9 % and 95.8 % identity with Orcos from *Bombyx mori* and *Heliothis virescens*, respectively [17-19].

### Phylogenetic analysis reveals orthology and paralogy with lepidopteran PRs

**Figure 1:**
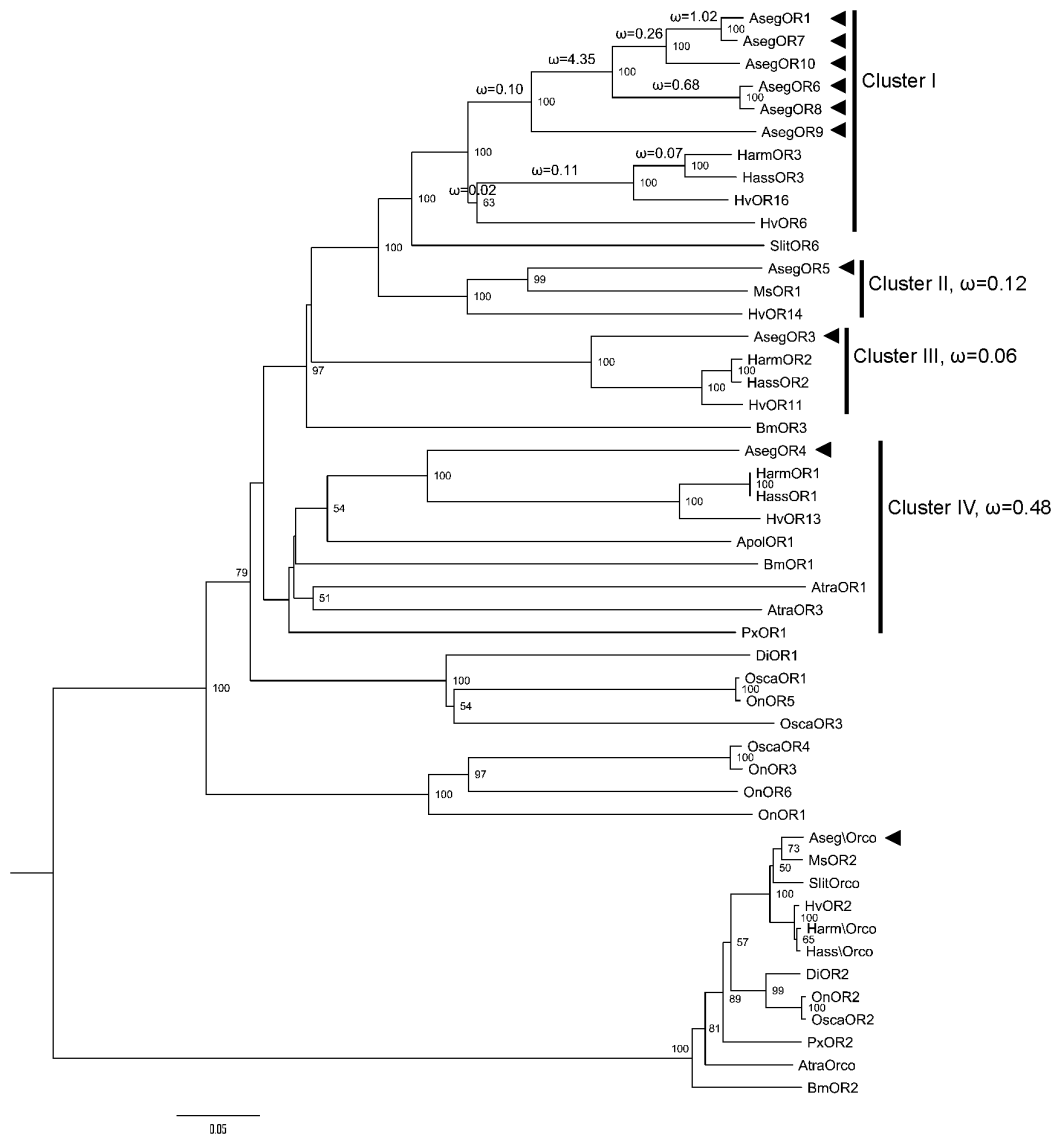
Neighbor-joining phylogenetic tree of AsegORs with functionally identified PR sequences in Lepidoptera. The tree was rooted with Orco lineage in Lepidoptera. Percentage bootstrap support (1000 replicates) values over 50 are shown at corresponding nodes. AsegORs and AsegOR\Orco are indicated with arrowheads. The nonsynonymous (dN) to synonymous (dS) substitution rate (ω) was labelled in the tree. Cluster II, III and IV have a uniform ω rate for all branches, as labelled at the right side, whereas Cluster I has various ω rate for all branches within the lineage, as labelled on the left on each branch. Genbank accession numbers of the protein sequences used in this phylogenetic tree were concluded in Table S2.

The nine putative PR genes in *A. segetum* were used to construct a phylogenetic tree together with other functionally identified lepidopteran PR genes, including those from *B. mori* (Bombycidae) [20,21], *H. virescens* (Noctuidae) [22,23], *Plutella xylostella* (Plutellidae), *Mythimna separata* (Noctuidae), *Diaphania indica* (Crambidae) [24], *Ostrinia scapulalis* (Crambidae) [25,26], *O. nubilalis* (Crambidae) [27], *Antheraea polyphemus* (Saturniidae) [28], *Spodoptera littoralis* (Noctuidae) [29], *Amyelois transitella* (Pyralidae) [30], *Helicoverpa armigera* (Noctuidae) and *Helicoverpa assulta* (Noctuidae) [31], based on their deduced amino acid sequences (Figure 1). The nine candidate *A. segetum* PRs fall into four orthologous clusters (assigned as Cluster I-IV), each of these containing representatives from other noctuids. Notably, the similar receptors AsegOR1 and AsegOR6-10, form a set of paralogues in Cluster I.

### Various selection pressures acting on lepidopteran PRs

In order to evaluate the selective pressures acting on each of the four clusters, we calculated the ratio of nonsynonymous to synonymous substitutions (dN/dS or ω) with branch models using PAML [8,32,33]. We first compared the log likelihoods (lnL) for the one ratio model M0 (assuming one ω ratio for all branches) and the free ratio model M1 (assuming one ω ratio for each branch) in likelihood ratio tests (Table S3). For Cluster II- IV, the one ratio model (M0) could not be rejected (*P*>0.01). The normalized ω ratios were significantly less than 1 for all of them (Figure 1), indicating purifying selection acting on all three clusters. However, for Cluster I model M1 gave a significantly better fit than model M0 (*P*<0.001), which suggested that different selective pressures were acting on different branches within this cluster. We then estimated the ω ratio of the branches within Cluster I. The branches in the lower expansion including the three *Heliothinae* species showed evidence of strong purifying selection (ω<<1, Figure 1). For the upper expansion, the branch leading to the six paralogues in *A. segetum* was under purifying selection (ω=0.10), whereas after duplication, the genes seemed to be under relaxed selection constraint, with strong evidence of positive selection at some branches (ω>1, Figure 1).

We noticed that the largest ω value was on the branch that separates AsegOR9 from the others, and the second largest ω value was on the branch that separates AsegOR1 and AsegOR7 from AsegOR10. In order to examine the positively selected sites in this branch of genes, we first confirmed the existence of positive selection sites in the genes by comparing the likelihood ratio of M0 (one ratio) and M3 (discrete) [32,34]. The discrete model M3 was significantly better fit than M0 (*P*<<0.001), which indicated variable ω value at the sites across the gene. For model M3, 37.6% of all sites had a ω value greater than 1. We next examined the positively selected sites computed by the naive empirical Bayes (NEB) under M3 [35], and found 12 sites with posterior probabilities > 95%: 63C, 65V and 67K in transmembrane domain 1 (TM1), 114M, 123Y and 134L in intracellular loop 1 (IC1), 158W in TM3, 227V in TM4, 268D and 283L in IC2, 385K and 386E in IC3.

### PRs from different clusters specifically responded to different sex pheromone components

We used two-electrode voltage clamp recording on *Xenopus* oocytes co-expressing each of the PRs with Orco to identify their ligand profiles. Except for the four pheromone components and two behavioural antagonists, we also included decyl acetate (10:OAc) as a stimulus for the oocytes, a component in the female sex pheromone gland extract that could elicit electroantennogram (EAG) activity, and early on was considered to be essential for full behavioural activity in the laboratory behavioural bioassay [11]. Thus seven chemicals were applied to the injected oocytes successively.

**Figure 2:**
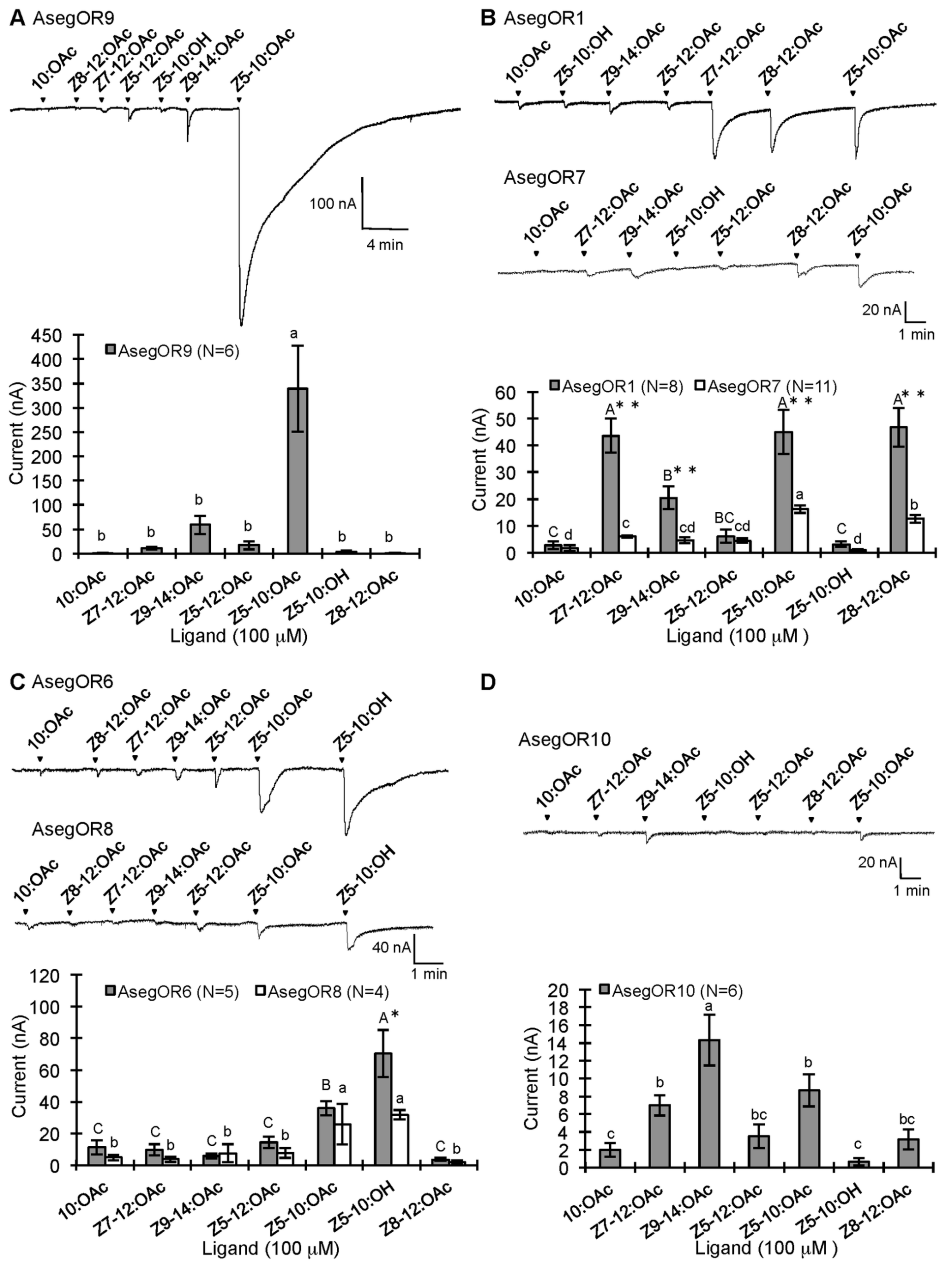
Distinct response profiles of the six AsegOR paralogues from Cluster I. Response profiles of (A) AsegOR9, (B) AsegOR1 and AsegOR7, (C) AsegOR6 and AsegOR8, (D) AsegOR10. The upper part of each panel indicates current trace of injected oocyte upon successive exposures to 100 μM stimuli. Each chemical was applied at the time point indicated by arrowheads for 20 s. The lower part of each panel indicates mean values ± SE of the stimulated currents in nA. Number of replicates for each receptor is indicated in the legend. Letters above bars represent significant level at p <0.05 (one-way-ANOVA followed a LSD test); single or two asterisks in (B) and (C) represent different sensitivities of two receptors to same ligand (p <0.05 or p<0.01 respectively, t-test).

**Figure 3:**
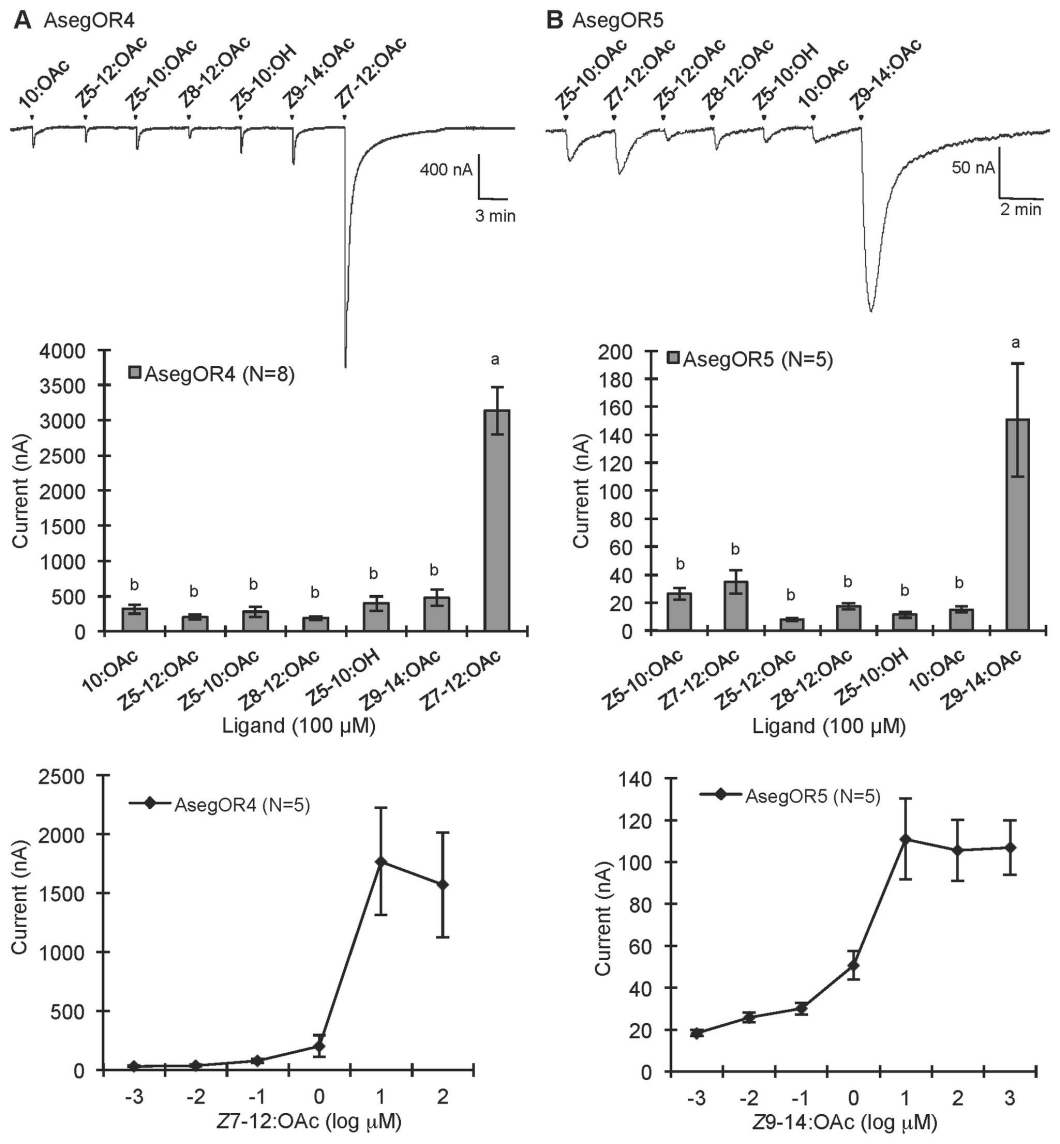
Response profiles and dose responses of (A) AsegOR4 and (B) AsegOR5. The upper part of each panel indicates current trace of injected oocyte upon successive exposures to 100 μM stimuli. Each chemical was applied at the time point indicated by arrowheads for 20 s. The middle part of each panel indicates mean values ± SE of the stimulated currents in nA, one-way-ANOVA followed a LSD test, p <0.05. The lower part of each panel indicates the responses of the receptors at different doses of ligand. Number of replicates for each receptor is indicated in the legend.

**Figure 4:**
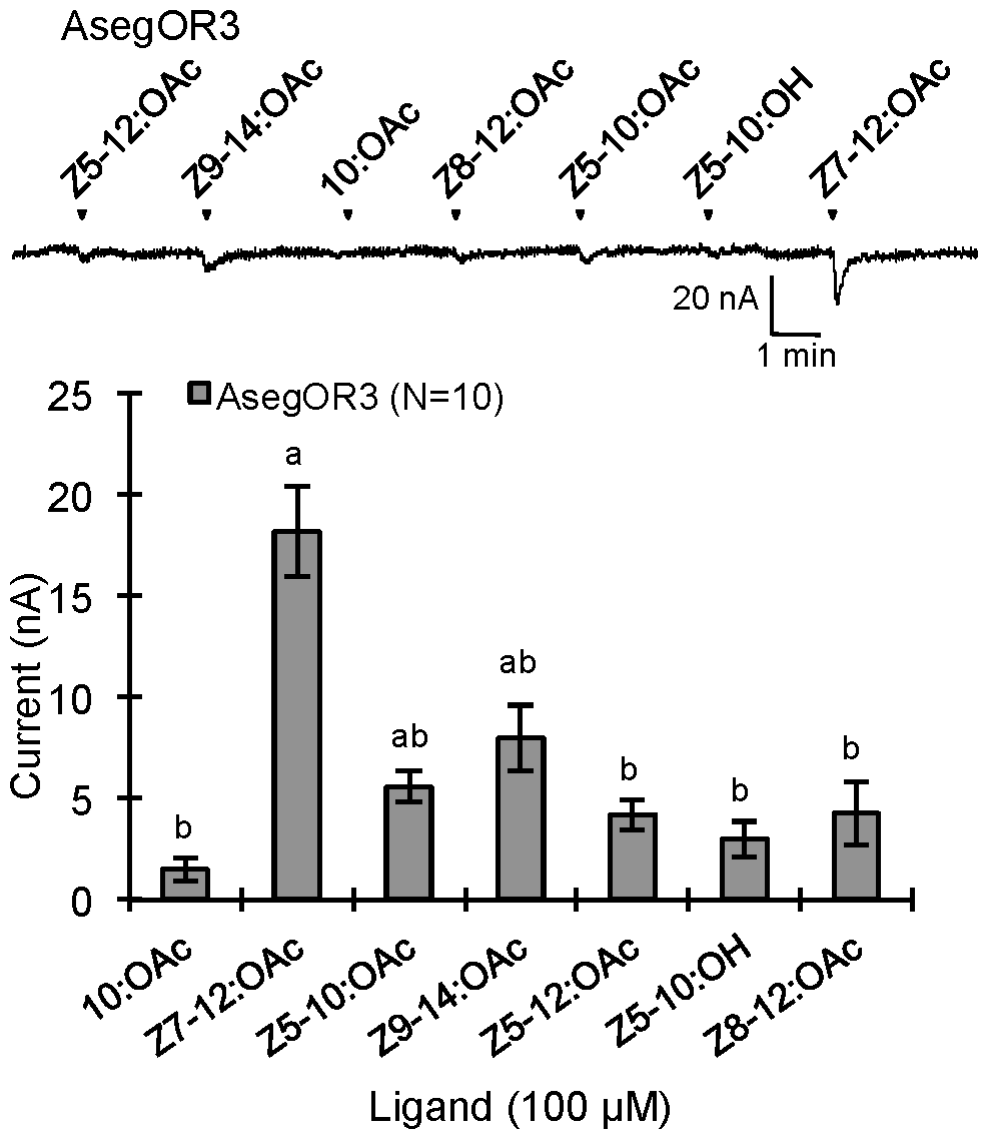
Response profiles and dose responses of AsegOR3. The upper part indicates current trace of injected oocyte upon successive exposures to 100 μM stimuli. Each chemical was applied at the time point indicated by arrowheads for 20 s. The lower indicates mean values ± SE of the stimulated currents in nA, one-way-ANOVA followed a LSD test, p <0.05. Number of replicates for each receptor is indicated in the legend.

AsegOR9, AsegOR4 and AsegOR5 occurred in different clusters of orthologues, and each showed specific responses to different pheromone components. The oocytes co-expressing AsegOR9 (Cluster I) and Aseg\Orco robustly and specifically responded to *Z*5-10:OAc, the behaviourally most important pheromone component in *A. segetum* (Figure 2A). AsegOR4 (Cluster IV) was specifically responsive to the pheromone component *Z*7-12:OAc, with a large magnitude (up to several µA at 100 µM ligand concentration) and high sensitivity response (Figure 3A). AsegOR5 (Cluster II) showed the largest response to *Z*9-14:OAc, a much smaller response to *Z*7-12:OAc and marginal responses to other tested compounds (Figure 3B). AsegOR3, which appeared in Cluster III, showed very small responses to all the tested compounds (Figure 4), albeit with Z7-12:OAc standing out as the most active one.

### Paralogous PRs from Cluster I differ dramatically in ligand selectivity and sensitivity

**Figure 5:**
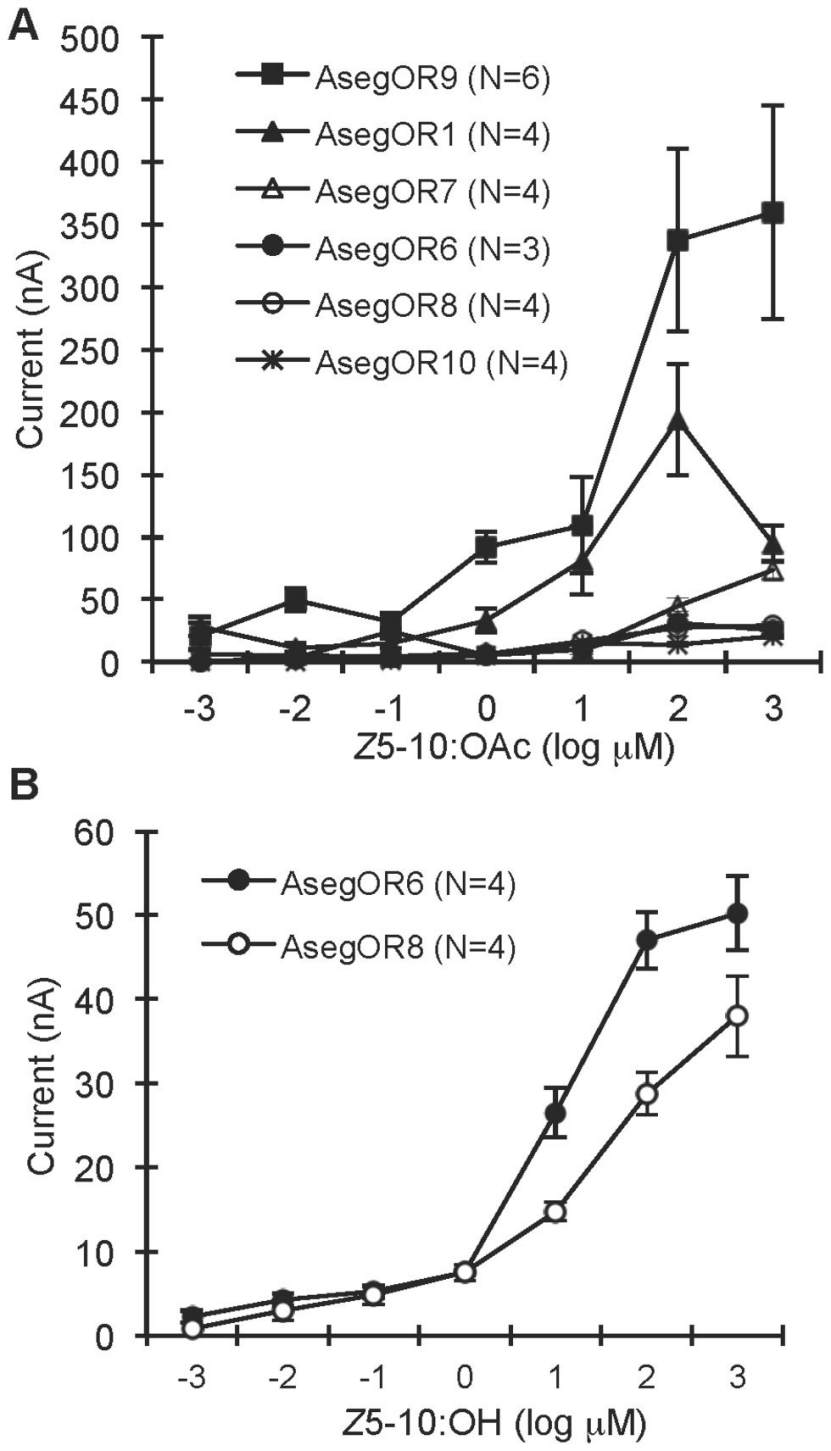
The six AsegOR paralogues from Cluster I vary in ligand sensitivity. (A) The dose responses of the six paralogues to the pheromone component *Z*5-10:OAc. (B) The dose responses of AsegOR6 and AsegOR8 to the behavioural antagonist *Z*5-10:OH. Number of replicates for each receptor is indicated in the legend.

Cluster I contains six paralogues including AsegOR9 showing the high and specific response to *Z*5-10:OAc as previously mentioned. However, the other five paralogues exhibited different ligand selectivities and sensitivities. Oocytes co-expressing AsegOR1 and Aseg\Orco also responded to *Z*5-10:OAc but more weakly and with lower specificity than AsegOR9. Thus AsegOR1 was broadly tuned, with similar responses to Z5-10:OAc, *Z*7-12:OAc, and the antagonist *Z*8-12:OAc (Figure 2B). Co-expression of another paralogue, AsegOR6, with Aseg\Orco produced oocytes that showed the strongest response to another antagonist *Z*5-10:OH, and a weaker response to *Z*5-10:OAc (Figure 2C). AsegOR7 has 97.7 % sequence identity (10 aa differences) to AsegOR1 and a similar response spectrum, albeit with much lower sensitivity (Figure 2B). Similarly, AsegOR8 was much less sensitive than AsegOR6, although they share 98.4% sequence identity (7 aa differences) and have the same response spectrum (Figure 2C). AsegOR10 responded to *Z*9-14:OAc and *Z*5-10:OAc but with low sensitivity (Figure 2D). It is worth mentioning that despite the fact that the sensitivity of receptors AsegOR7 and AsegOR10 is much lower than the sensitivity of AsegOR1, AsegOR6 and AsegOR9, they still displayed distinguishable and selective responses to certain ligands. Different sensitivities of the six paralogues to the main pheromone component *Z*5-10:OAc are shown by dose response curves in Figure 5A. Two of them (AsegOR6 and AsegOR8) showed strong responses to Z5-10:OH (Figure 5B).

## Discussion

Due to the high divergence of insect OR sequences, most of them were previously cloned based on genomic sequences, transcriptome analysis or cDNA library screening. However, the relatively high degree of amino acid sequence identity among PR genes in lepidopteran insects makes it possible to identify new PR gene sequences by degenerate primer based RT-PCR and RACE PCR [24,30,36]. The degenerate primers we used in this study were obviously effective to explore PRs from *A. segetum*. PR genes in moths show a male-biased expression in antennae, as demonstrated in *B. mori*, *H. virescens* and other species [20-22,24-29,31]. These genes form a subgroup in the phylogenetic tree of lepidopteran olfactory receptors. The clustering of the nine candidate *A. segetum* receptors in the PR subfamily suggests a similar expression pattern although we did not explore this explicitly. The specific receptors for pheromone components *Z*5-10:OAc, *Z*7-12:OAc and *Z*9-14:OAc, as well as receptors for behavioural antagonist *Z*5-10:OH were identified. No specific receptor for the minor pheromone component *Z*5-12:OAc was found among the nine PRs, but several PRs gave a minor response to this compound. It’s unclear whether there is actually one specific *Z*5-12:OAc receptor because in previous *in vivo* single sensillum recordings, three different *Z*5-12:OAc-responsive receptors also responded to *Z*5-10:OAc, *Z*5-10:OH or *Z*7-12:OAc [12,13]. Receptors AsegOR3, AsegOR7, AsegOR8 and AsegOR10 showed weak responses to the pheromone and analogs. Such receptors might not be behaviourally important, whereas those more responsive receptors, i.e. AsegOR1, AsegOR4, AsegOR5, AsegOR6 and AsegOR9 should play a crucial role in the male detection of pheromone components.

**Figure 6:**
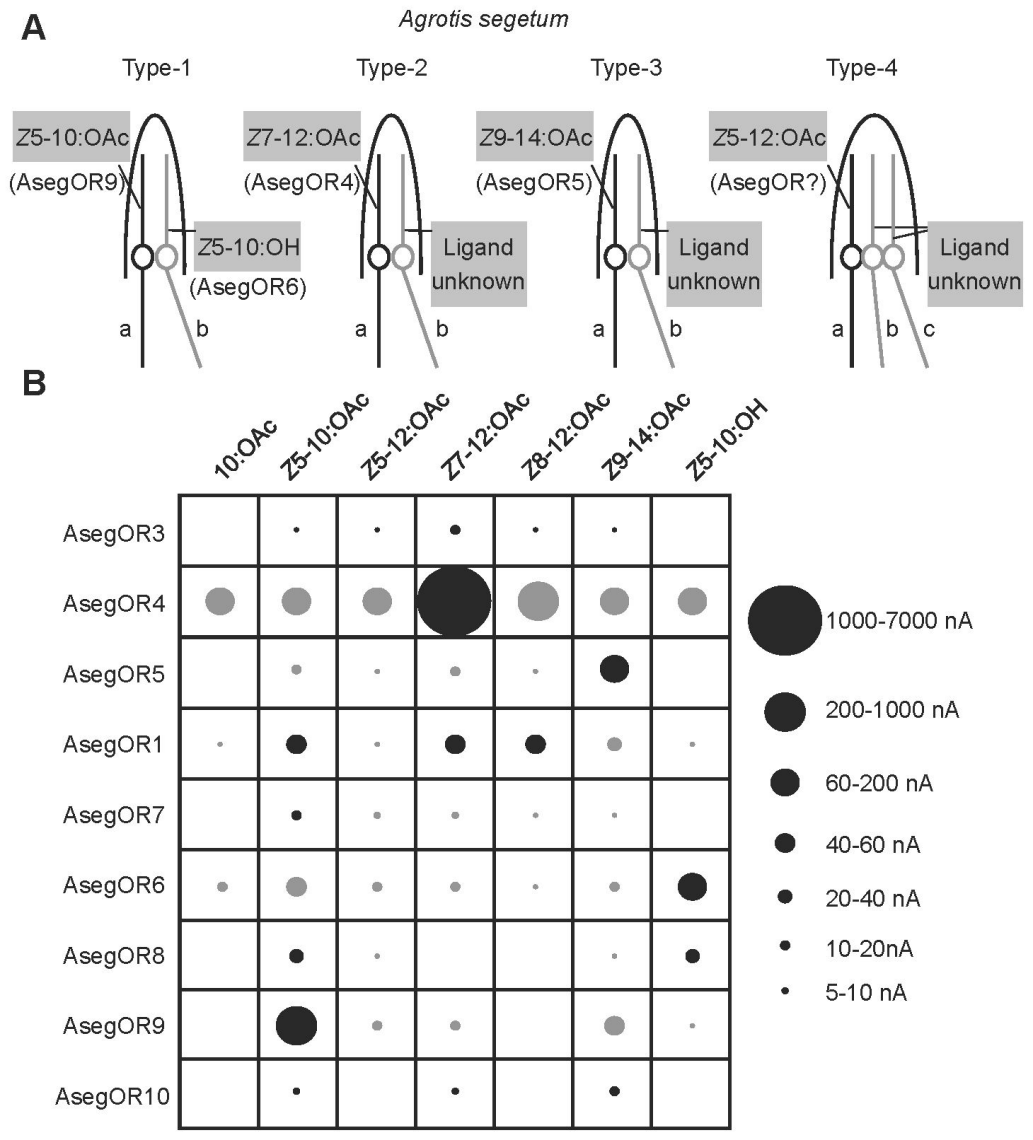
Summary of the response profiles of AsegOR1, AsegOR3-10 and the putative locations of PRs in the ORNs. (A) Representation of ORNs in trichoid sensilla of *A. segetum*, depicting suggested associations between PRs and ORNs. The different types of ORNs were identified in previous single-sensillum recordings [11-13]. (B) The response profiles of AsegOR1 and AsegOR3-10 in the presence of seven test compounds at a dose of 100 μM. Circles of different sizes represent the response magnitudes. Black circles indicate the responses of the most active ligand(s) of each receptor, whereas grey circles indicate responses evoked by other stimuli.

Extensive knowledge on the response spectra of *A. segetum* ORNs obtained from previous studies makes it possible to propose the assignment of PRs to ORNs (Figure 6A). The large spike amplitude neuron (neuron a) in sensillum Type-1, Type-2, and Type-3 are specifically tuned to pheromone components *Z*5-10:OAc, *Z*7-12:OAc and *Z*9-14:OAc, respectively [11,13], corresponding to the ligand profiles of AsegOR9, AsegOR4 and AsegOR5. We therefore suggest that these PRs are expressed in the ORNs mentioned (Figure 6). The response spectra of the different *Z*5-10:OAc-responsive paralogues to other pheromone components are also in agreement with the distinct subtypes identified in *Z*5-10:OAc ORNs [12]. The small spike magnitude neurons in sensillum Type-1 (neuron Type-1b) are tuned to *Z*5-10:OH and/or *Z*5-12:OAc. AsegOR6 is mainly responsive to the antagonist *Z*5-10:OH, although it also shows a small response to *Z*5-10:OAc, so we putatively locate AsegOR6 in ORN Type-1b. These assignments remain putative until the proposed pattern has been confirmed by for instance *in situ* hybridization.

The specific receptors differ in their response characteristics with the *Z*7-12:OAc receptor AsegOR4 being the most sensitive and with the largest response magnitude. We suppose this is real and not an artefact of the *in vitro* expression system as the same quality and quantity of the injected genes were ensured as far as possible during the experiment. Rather it is likely to reflect the inherent properties of receptors because the different response characteristics of *A. segetum* receptors are evident also in previous *in vivo* single sensillum recordings, in which different types of ORNs gave different spike frequencies to stimuli at the same dose [12,13]. We also notice that although AsegOR4 shows very large response to *Z*7-12:OAc, its response to some of the other compounds are actually larger than the response recorded in their respective specific receptors (Figure 6B). However, when the AsegOR4 injected oocytes were pre-exposed to *Z*7-12:OAc, the responses to the other compounds were reduced (Figure S1). In addition, the ligand selectivity of AsegOR4 might be further modified *in vivo* by other proteins like pheromone binding proteins (PBPs) and sensory neuron membrane proteins (SNMPs).

In addition to the specific receptors for each of the main pheromone components, we also found some broadly tuned receptors (e.g. AsegOR1). The existence of both specific and broadly tuned PRs is probably the case also in other moth species such as *Ostrinia* spp. [26,27]. One can at this point just speculate about the adaptive significance of this pattern, but we suggest that in combination with specific pheromone receptors, the broadly tuned pheromone receptors may provide male moths with the means to monitor variable female pheromone blends. The purifying selection acting on most of the PR orthologous clusters (Cluster II- IV in Figure 1) taken together with the broad response spectra of some other PRs, is in agreement with a conceptual model based on the asymmetric tracking hypothesis, according to which males experience stronger sexual selection than females and thus male pheromone response should be broad enough to track variation in the pheromone signal produced by females [8,27,37]. The receptors for the conspecific pheromone components are generally under purifying selection but a broader response profile of a subset of receptors may be an adaptation or preadaptation to allow males to track changes in female pheromone production.

Most interestingly, we identified one set of six paralogues in *A. segetum* that differ dramatically in ligand selectivity and sensitivity. Evolution of sex pheromone receptors through gene duplication has been suggested by previous studies on pheromone receptors in *H. virescens* where phylogenetic analysis showed that the four genes HvOR6, HvOR14, HvOR15, and HvOR16 are more closely related to each other than to any other Hv ORs [38]. Genetic mapping confirmed that they form a tightly linked cluster of duplicated genes [7]. These studies point to gene duplication as the mechanism underlying the functional evolution of pheromone receptor system. The discovery and functional identification of PR paralogues in our study provides further support to this hypothesis. Furthermore, we argue that the relaxed selection constraint or positive selection after duplication (as in Cluster I, Figure 1) may allow the six paralogues to accumulate beneficial mutations and thus gain functional divergence. The largest ω value on the branch that separate AsegOR9 from the other paralogues suggests that the amino acid changes at the positively selected sites are associated with the evolution of *Z*5-10:OH receptors (AsegOR6 and AsegOR8) and receptors for *Z*8-12:OAc and *Z*7-12:OAc (AsegOR1 and AsegOR7) from the ancestral *Z*5-10:OAc receptor AsegOR9 after gene duplication. This could be tested by future mutagenesis experiments, as in the example of *Ostrinia* species, a single mutation in a PR that is broadly responsive and under positive selection could narrow its specificity [10].

Different selective pressures on pheromone receptors were previously demonstrated in *Ostrinia* species [10] and this might be important characteristic for the evolution of the pheromone receptor multigene family. On one hand the overall purifying selection on most clusters results in the conserved function of the genes throughout related species; on the other hand, the relaxed selective constraint or positive selection on certain clusters might be important modulating the species-specific sex pheromone recognition, allowing fixation of advantageous mutations in the genes and modifying their ligand selectivity and sensitivity. This is in accordance with our observation (Table 2) that the paralogous PRs with high sequence similarities in Cluster I under relaxed selective constraint or positive selection differ dramatically in ligand selectivity and sensitivity, whereas the orthologues with relatively low sequence similarities in Cluster II- IV under purifying selection tend to be functionally more conserved than paralogues (e.g., PxOR1 and HvOR13 have only 43.4% sequence identity, but they share the same ligand *Z*11-16:Ald [22-24]).

**Table 2:** Summary of response profiles of functionally characterized lepidopteran PRs in Cluster I-IV.

**Clusters**	**Receptor**	**Main ligand**
**Ⅰ**	HvOR16	*Z*11-16:OH
	HvOR6	*Z*9-14:Ald
	HarmOR3	*Z*11-16:OH
	HassOR3	*Z*9-14:Ald
	AsegOR9	*Z*5-10:OAc
	AsegOR6	*Z*5-10:OH, *Z*5-10:OAc
	AsegOR1	*Z*5-10:OAc, *Z*7-12:OAc, *Z*8-12:OAc
	AsegOR7	Similar to AsegOR1, lower sensitivity
	AsegOR8	Similar to AsegOR6, lower sensitivity
	AsegOR10	Small response to *Z*9-14:OAc, *Z*5-10:OAc
**Ⅱ**	HvOR14	*Z*11-16:OAc
	MsOR1	*Z*11-16:OAc
	AsegOR5	*Z*9-14:OAc
**Ⅲ**	HvOR11	Ligand unknown
	HarmOR2	Broadly tuned
	HassOR2	Broadly tuned
	AsegOR3	Low sensitivity to all the tested compounds
**Ⅳ**	HvOR13	*Z*11-16:Ald
	HarmOR1	*Z*11-16:Ald
	HassOR1	*Z*11-16:Ald
	AtraOR3	*Z*11-16:Ald
	PxOR1	*Z*11-16:Ald
	AtraOR1	*Z*11,*Z*13-16:Ald
	ApolOR1	E6,*Z*11-16:Ald
	BmOR1	E10,*Z*12-16:OH
	AsegOR4	*Z*7-12:OAc

In conclusion our study of pheromone receptors in the turnip moth not only provides a complete examination of the response characteristics of the family of PRs in a noctuid moth but also suggest a possible scenario for pheromone receptor functional evolution and provides the basis for further investigations of the sequence differences determining clear-cut differences in ligand selectivity among receptor homologues.

## Materials and Methods

### Ethics statement

The care and use of *Xenopus laevis* frogs in this study were approved by the Swedish Board of Agriculture.

### Insect material

The *A. segetum* moths used in this study were obtained from our continuous culture at Department of Biology, Lund University. Larvae were reared on an semi-synthetic bean-based diet [39], and kept at 25 °C under the light–dark cycle of 16:8. The antennae were dissected from 1- to 3-day old virgin male adults that had been kept separated from females since the pupal stage.

### RNA preparation and first strand cDNA synthesis

Adult male moth antennae (20–40 pairs) were dissected under a stereomicroscope. The dissected materials were homogenized in Trizol reagent (Invitrogen, Carlsbad, CA, USA) using a glass grinder on ice. Total RNA isolation was performed according to the manufacturer’s instructions immediately after dissection. First-strand cDNA was synthesized from 1 μg total RNA using ThermoScript RT-PCR System (Invitrogen, Carlsbad, CA, USA), with 1μl of oligo-dT primer in a 20-μl reaction mixture.

### Sequencing of PRs and *Orco* cDNA fragments

Degenerate primers were designed based on the lepidoptera pheromone receptor and Orco sequences (Table S1), and the forward and reverse primers were used in all possible combinations. Two rounds of PCR were performed with a Veriti 96-well Thermal Cycler (Applied biosystems, Carlsbad, CA, USA) using DreamTaq Green DNA Polymerase

(Fermentas AB, Sweden). The first round PCR started with an initial denaturation at 95 °C for 3 min; followed by 40 cycles of 1 min at 95 °C, 1 min at 40 °C, 1 min at 72 °C, and final extension at 72 °C for 5 min. 1-µl product of the first round PCR was used as template in a

25-µl reaction mixture for second round PCR, which consisted of an initial denaturation at 95 °C for 3 min, followed by 45 cycles of the same PCR cycling parameters as those of the first round of PCR.

PCR products with expected size were gel purified using Wizard SV Gel and PCR Clean-Up System (Promega, Madison, WI, USA), and the fragments were ligated into pTZ57R/T vector (Fermentas AB, Sweden). After transformation into TOP10 competent cells (NEB, Ipswich, MA, USA), the recombinant clones were picked by colony PCR and sequenced using universal M13 primers and the BigDye terminator cycle sequencing kit v1.1 (Applied Biosystems, Carlsbad, CA, USA). Sequencing products were EDTA/ethanol-precipitated, dissolved in formamide and loaded for analysis on a capillary 3130xl Genetic analyzer (Applied Biosystems, Carlsbad, CA, USA).

### RACE amplification and sequencing of full-length cDNAs

Based on the cDNA fragment sequences, several pairs of primers were designed for RACE PCR (Table S1). The 5’ and 3’ RACE were performed using BD SMART RACE cDNA Amplification Kit (Clontech, Mountain View, CA, USA) following the manufacturer’s manual. The RACE PCR program included initial denaturation at 94 °C for 2 min, followed by 5 cycles of 30 s at 94 °C and 3 min at 72 °C; 5 cycles of 30 s at 94 °C, 30 s at 70 °C, 3 min at 72 °C; 28 cycles of 30 s at 94 °C, 30 s at 68 °C, 3 min at 72 °C; and a final extension step of 7 min at 72 °C. The RACE PCR products were subcloned into pTZ57R/T vector and sequenced. The full-length cDNA sequences were assembled from the cDNA fragments and the sequences from 5’ and 3’-RACEs.

According to the assembled full-length cDNA sequences, several pairs of primers with restriction sites were designed for amplifying the full-length cDNAs with *PfuUltra* II Fusion HS DNA Polymerase (Agilent Technologies Inc., Santa Clara, CA, USA) (Table S1). The PCR program included initial denaturation at 95 °C for 1 min, followed by 40 cycles of 20 s at 95 °C, 20 s at 55 °C, 1 min at 72 °C, and a final extension at 72 °C for 3 min. PCR products with expected size were gel purified and subcloned into pCS2+ vectors (kindly provided by Dr. Ying Liu, Institute of Biophysics, Chinese Academy of Sciences). Recombinant clones were picked by colony PCR and sequenced.

### Sequence and phylogenetic analyses

The sequence analyses were performed by Vector NTI Advance 10 software (Invitrogen, Carlsbad, CA, USA). Deduced amino acid sequences were aligned with the PR protein sequences that had been functionally identified from other lepidopteran species by BioEdit [40]. Genbank accession numbers of all the PR protein sequences are presented in Table S2. The neighbour-joining method was employed to construct the phylogenetic tree by PAUP*4.0 [41]. The tree was rooted with the Orco lineage in lepidopterans. Bootstrap analysis (N = 1000) was performed. Estimation of the nonsynonymous (dN) to synonymous (dS) substitution rate (ω) was performed by the maximum likelihood method [42] using the Codeml program in the PAML 4.6 package [32].

### Chemicals

All acetates used in this study were from our laboratory stock and were overall >96% pure. *Z*5-10:OH was prepared by hydrolysis of *Z*5-10:OAc [43]. Stock solutions were prepared in dimethyl sulfoxide (DMSO) (Sigma-Aldrich Co., St. Louis, MO, USA), and diluted to indicated concentrations with Ringer’s buffer (96 mM NaCl, 2 mM KC1, 5 mM MgC1_2_, 0.8 mM CaCl_2_, 5 mM HEPES, pH 7.6) before experiment. Ringer's buffer containing 0.1% DMSO was used as a negative control.

### Oocyte injection and two-electrode voltage clamp recording

cRNAs of candidate PR genes and *Orco* were synthesized from the linearized recombinant plasmids with mMESSAGE mMACHINE Kit (Applied Biosystems, Carlsbad, CA, USA).

*X. laevis* (purchased from *Xenopus* Express France, Vernassal, Haute-Loire, France) oocytes were treated with 1.5 mg / mL collagenase (Sigma-Aldrich Co., St. Louis, MO, USA) in Oocyte Ringer 2 solution (82.5 mM NaC1, 2 mM KCl, 1 mM MgC1_2_, 5 mM HEPES, pH 7.5) at 20°C for 1-2 hr. Stage V-VII oocytes were co-injected with 50 ng each of candidate PR gene cRNA and *Orco* cRNA, then incubated in Ringer’s buffer supplemented with 550 mg/L sodium pyruvate and 100 μg/ml gentamicin at 18°C for 3-5 days [44].

Whole-cell inward currents were recorded by two-electrode voltage clamp with a TEC-03BF amplifier (npi electronic GmbH, Tamm, Germany) at the holding potential of -80 mV. Compounds used for stimulation were applied through a computer-controlled perfusion system to the chamber holding oocytes for 20 s at a rate of 2 ml/min, with extensive washing in Ringer’s buffer at a rate of 4 ml/min between applications that allowed the current to return to baseline. Data were collected and analyzed by Cellworks software (npi electronic GmbH, Tamm, Germany). Mean values of current ± SE of the oocytes responding to 100 μM stimuli were compared by One-Way-ANOVA followed a LSD test (significance level: p <0.05) with SPSS10.0.1 software (SPSS Inc., Chicago, IL, USA).

## Supporting Information

Figure S1 **Inhibitory effect of *Z*7-12:OAc on the response of AsegOR4 to other compounds.**
(PDF)

Table S1 **Primers used in this article.**
(PDF)

Table S2 **GenBank accession numbers of the PRs used in the phylogenetic tree.**
(PDF)

Table S3 **Variable selective pressures acting on PR orthologous clusters.**
(PDF)
