# Supplementary material for: Functional Evolution of a Multigene Family: Orthologous and Paralogous Pheromone Receptor Genes in the Turnip Moth, Agrotis segetum
Source: PLoS One. 2013 Oct 10;8(10):e77345. doi: 10.1371/journal.pone.0077345 (PMC3795068; doi:10.1371/journal.pone.0077345)

Figure S1. Inhibitory effect of *Z*7-12:OAc on the response of AsegOR4 to other compounds. (A) Current trace of the oocytes co-injected with AsegOR4 and AsegOrco upon successive stimuli, when *Z*7-12:OAc was used as the first stimulus. (B) Mean values ± SE of the stimulated currents in nA (N=4). Other compounds stimulated smaller response if they were applied to the oocytes immediately after the major ligand *Z*7-12:OAc. Single or two asterisks represents significant level of the difference (p <0.05 or p<0.01 respectively, t-test).

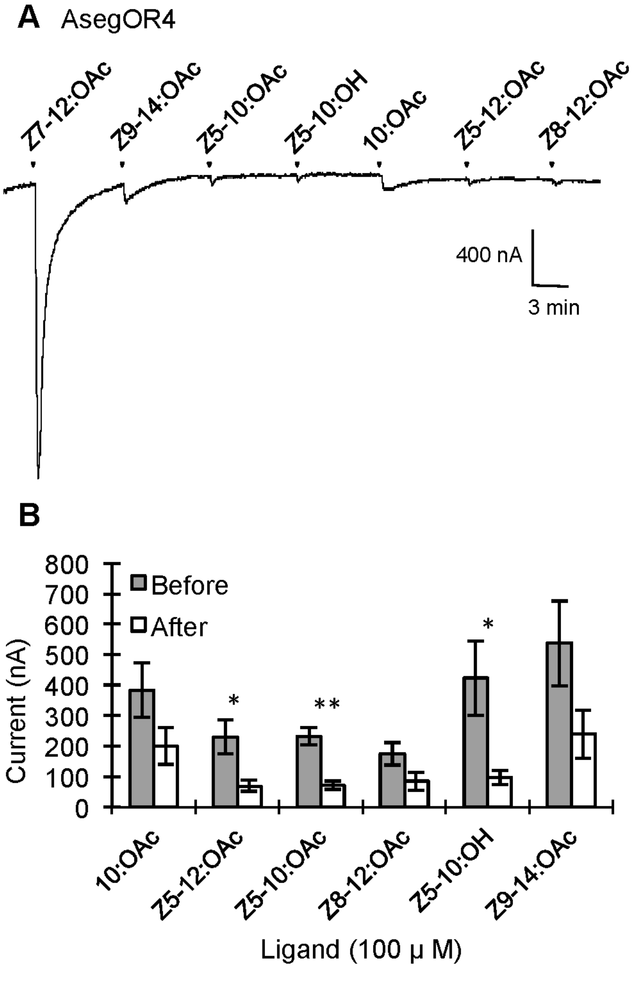

Supplement: Figure S1 — Inhibitory effect of Z7-12:OAc on the response of AsegOR4 to other compounds. (PDF) [file pone.0077345.s001.pdf]
