# Supplementary material for: Functional Evolution of a Multigene Family: Orthologous and Paralogous Pheromone Receptor Genes in the Turnip Moth, Agrotis segetum
Source: PLoS One. 2013 Oct 10;8(10):e77345. doi: 10.1371/journal.pone.0077345 (PMC3795068; doi:10.1371/journal.pone.0077345)
Supplement: Table S1 — Primers used in this article. (PDF) [file pone.0077345.s002.pdf]

Table S1. Primers used in this article.

Primer	Primer sequence (5’→3’)	
	**Primers for sequencing of PR cDNA fragments**	
A1	GGITGYYTIYTIYTIYTIGARTGYTC	S
A2	GGITGYATHYTIYTIYTIGARTGYTC	S
A3	TGYTGYYTIYTIYTIYTIGARTGYWC	S
B1	CARCARYTIATHCARYTITC	S
B2a	CAACAAYTNATHCAAATHTC	S
B2b	CAACAAYTNATHCAAATHAG	S
C1	TNCCDTGGGARTRYATGG	S
C2	TNCCDTGGGARKCYATGG	S
C3	TNCCDTGGGARAGYATGG	S
D1	ATNGANGCCATNGTIGTIACICC	AS
D2	NGYCATNGWYTGIACICC	AS
D3	ATCGCNGYCATNGAIGTIACICC	AS
E1	TAIGAIAWIGAIGTYTTYARDAT	AS
E2	TAIGAIADIGAIGTYTTDATDAT	AS
E3	TAIGAIADIGTIGTYTTYARDAT	AS
Orco5	ATHAARTAYTGGGTIGARMGICAYAARCA	S
Orco3	CATIGCYTTYTGRCAYTGYTGRCAIACDAT	AS
	**Primers for RACE**	
1L	GGACAAGGAGAACAGGAAAGTAGTGACC	S
1R[a]	AGACCCAGGGCCTTCACATGTACCG	AS
2L	TGAATTGGTAGGAAGTGAGAGTGCAAAA	S
2R	TAAGAAGGCCACCACTTTCCTGTTCTCC	AS
3L	CAAGAACCGCAGGACAGCCCTCATT	S
3R	ACGTGAATCGGCTCCTGAACCTTGA	AS
4L	GGAGCAGGTGCAGCGGTCCATGA	S
4R	GTGAGCATTCCCATGGCCTTCAGGT	AS
5L	GTATTAGTGACAGATTGCAAGGCGCTGT	S
5R	TCACAGTTCGTCTGTTCCTCGTATCCA	AS
6L	CGAAGAACAGGAAAGTGGTGGCCTTC	S
6R	CCCAGCGCCTTCACATGTATTGGATT	AS
7L	GGACACGAAGAACAGGAAAGTGGTGACC	S
8L	GGAAGTACGAGTGATAAACTGAAGGATGC	S
8R	TTGAGTCCATATATTGCCATGGAACACC	AS
9L	GCCATGGCAAAGTATGGACAATTTGAAC	S
9R	AGGTCACCATGTTCCTGTTCAAATTGTC	AS
OrcoL	GTTCCACATGCTGGTGTCAACGATCA	S
OrcoR	GAGCCGTCGTACCACTGGCAGGAAT	AS
	**Primers for construction of recombinant pCS2+ vectors**	
G1res5	*CG*GAATTC**GCCACC**ATGACTTTACGACAATTCCTTTTCGAAAATGAATCGGTT	S
G1res3	*CCG*CTCGAGCTACATGCTGCGTAGAAAGGTGAAGTAAGACAAC	AS
G2res5[b]	*CG*GAATTC**GCCACC**ATGACTTTACGACAATTCCTTTTCGAAAACGAACCA	S
G2res3[b]	*CCG*CTCGAGCTACTTGCTGCGTAGAAAGGCGAAGTAAGACATTGAA	AS
G3res5	*CG*GAATTC**GCCACC**ATGCTCGCTAGAATTGAAAAACTTGCTGG	S
G3res3	*CCG*CTCGAGTTAACTATCGAAAGTACGCAGGAAAGC	AS
G4res5	*CGC*GGATCC**GCCACC**ATGAAATTATTCTCCGATCTGTCTGACCTGG	S
G4res3	*CG*GAATTCTTATTCATCTTCCTCTGCAACTGTTTGTAGC	AS
G5res5	*CGC*GGATCC**GCCACC**ATGCCCTCTTCTAGTTTACGCGAT	S
G5res3	*CCG*CTCGAGTCAATTGCTTCTCAGAAAAATGAAGTAAG	AS

G6res3	*CCG*CTCGAGCTACTTGCTGCGTAGAAATGTGAAGTAAGACATTGAT	AS
G7res5	*CG*GAATTC**GCCACC**ATGACTTTACGACAATTCCTTTTCGAAAACGAATCGGTC	S
G7res3	*CCG*CTCGAGCTACATGCTGCGTAGAAAGGTAAAGTAAGACAGT	AS
G9res5	*CG*GAATTC**GCCACC**ATGTCTTTACGAAAATTCCTTTTAGAAAACGAAGCCGTA	S
G9res3	*CCG*CTCGAGCTACATGCTGCGTAGAAAGGTGAAGTAAGACAT	AS
Orco-res5	*CGC*GGATCC**GCCACC**ATGATGACCAAAGTGAAGGCCCAGG	S
Orco-res3	*CCG*CTCGAGTTACTTGAGTTGCACCAACACCATG	AS

S: sense primer; AS: antisense primer. The underlined indicate restriction recognition sites, the italic indicate bases flanking the recognition sequences, and the bold indicate Kozak sequence. [a]This primer was used for both *AsegOR1* and *7*, as they were designed at identical regions. [b]These were common primers used for both *AsegOR2* and *8*. G2res5 was also used as 5' primer for *AsegOR6*.
