# Supplementary material for: Functional Evolution of a Multigene Family: Orthologous and Paralogous Pheromone Receptor Genes in the Turnip Moth, Agrotis segetum
Source: PLoS One. 2013 Oct 10;8(10):e77345. doi: 10.1371/journal.pone.0077345 (PMC3795068; doi:10.1371/journal.pone.0077345)
Supplement: Table S2 — GenBank accession numbers of the PRs used in the phylogenetic tree. (PDF) [file pone.0077345.s003.pdf]

Table S2. GenBank accession nos of the PRs used in the phylogenetic tree.

PR protein name	Accession no.
Agrotis segetum	
AsegOR1	KC526965
AsegOR2	KC526966
AsegOR3	KC526967
AsegOR4	KC526968
AsegOR5	KC526969
AsegOR6	KC526970
AsegOR7	KC526971
AsegOR8	KC526972
AsegOR9	KC526973
Heliothis virescens	
HvOR11	CAG38112.1
HvOR13	CAG38114.1
HvOR14	CAG38115.1
HvOR16	CAG38117.1
Bombyx mori	
BmOR1	BAD69584
BmOR3	BAD89567
Plutella xylostella	
PxOR1	BAG71420.1
Mythimna separata	
MsOR1	BAG71414.1
Diaphania indica	
DiOR1	BAG71417.1
Ostrinia scapulalis	
OscaOR1	BAH57975.1
OscaOR3	BAI66604.1
OscaOR4	BAI66605.1
Ostrinia nubilalis	
OnOR1	ADB89178.1
OnOR3	ADB89180.1
OnOR5	ADB89182.1
OnOR6	ADB89183.1
Antheraea polyphemus	
ApolOR1	CBH19582.1
Spodoptera littoralis	
SlitOR6	ACL81183.1
Amyelois transitella	
AtraOR1	AFP54146.1
AtraOR3	AFP54147.1
Helicoverpa armigera	
HarmOR1	ACS45304
HarmOR2	ACS45305
HarmOR3	ACS45306
Helicoverpa assulta	
HassOR1	ACS45307
HassOR2	ACS45308
HassOR3	ACS45309

**Orco lineage**	
Aseg\Orco	KC526964
HvOR2	CAD31851.1
BmOR2	BAD69585
PxOR2	BAG71421.2
MsOR2	BAG71415.1
DiOR2	BAG71418.1
OscaOR2	BAH57973.1
OnOR2	ADB89179.1
Slit\Orco	ABQ82137.1
Atra\Orco	AFP54145.1
Harm\Orco	HQ186284
Hass\Orco	HQ186285
