# Supplementary material for: Functional Evolution of a Multigene Family: Orthologous and Paralogous Pheromone Receptor Genes in the Turnip Moth, Agrotis segetum
Source: PLoS One. 2013 Oct 10;8(10):e77345. doi: 10.1371/journal.pone.0077345 (PMC3795068; doi:10.1371/journal.pone.0077345)
Supplement: Table S3 — Variable selective pressures acting on PR orthologous clusters. (PDF) [file pone.0077345.s004.pdf]

Table S3. Selective pressures acting on PR orthologous clusters.

Cluster	n	InL M0	InL M1	df	*P* value
**I**	10	-6515	-6457	17	*P*<0.001
**II**	3	-3830	-3837	3	*P*>0.01
**III**	4	-3332	-3326	5	*P*>0.01
**IV**	9	-9338	-9329	15	*P*>0.01

Selective pressures acting on four orthologous clusters were tested by the branch-specific models in codeml (PAML 4.6 package). The log likelihoods (lnL) for branch models M0 (assuming one nonsynonymous to synonymous substitution rate for all branches) and M1 (assuming one nonsynonymous to synonymous substitution rate for each branch) were compared in likelihood ratio tests (LRT). *P* values were determined by comparison of the LRT statistics to a $\chi 2$ table using the relevant number for degrees of freedom. M0 model is better fit for Clusters II-IV, and M1 model is better fit for Cluster I. n, number of sequences; df, degrees of freedom.
